# Editorial: Subcellular computations and information processing

**DOI:** 10.3389/fnsyn.2023.1169671

**Published:** 2023-03-07

**Authors:** Tomoe Ishikawa, Ayako Wendy Ishikawa, Athanasia Papoutsi, Asami Tanimura, Keisuke Yonehara

**Affiliations:** ^1^The Picower Institute for Learning and Memory, Massachusetts Institute of Technology, Cambridge, MA, United States; ^2^The Department of Brain and Cognitive Sciences, Massachusetts Institute of Technology, Cambridge, MA, United States; ^3^Department of Physiology, Keio University School of Medicine, Tokyo, Japan; ^4^Institute of Molecular Biology and Biotechnology, Foundation for Research and Technology – Hellas, Heraklion, Greece; ^5^The Department of Biomedicine, Aarhus University, Aarhus, Denmark; ^6^Department of Gene Function and Phenomics, National Institute of Genetics, Mishima, Japan; ^7^Department of Genetics, The Graduate University for Advanced Studies (SOKENDAI), Mishima, Japan

**Keywords:** subcellular computation, dendrite, cell-type, neuromodulators, synapse

A neuron receives thousands of synaptic inputs at its dendritic branches, integrates them both locally and at somatic level, and sends output to downstream neurons *via* its axon terminals. In this process, the somatic outcome is actively modified by the morphology of the dendritic branches, the expression of channels along the somato-dendritic axis, and spatiotemporal patterns of activation of the synaptic inputs. Recently, it has been suggested that axonal output is also locally regulated along its arbors contrary to the conventional idea of digital-like uniformity. However, we still do not fully understand the nature or logic of the subcellular computations carried out within a single neuron.

The aim of this Research Topic is to bring together publications from a wide range of topics on subcellular computation to facilitate our understanding of how the network activity organizes and modifies the spatiotemporal pattern of synaptic inputs and outputs. Particularly, this Research Topic focuses on the processes by which a single neuron integrates several synaptic inputs from upstream neuron assemblies and transmits a variety of output patterns to downstream neuron assemblies. We successfully gathered two original publications and two review articles written by 10 authors in total. The papers cover the topic of subcellular computation in various species, including mice, rats, ferrets, and birds.

Two original papers focused on the subcellular computation conducted in single neurons using computational models. Meissner-Bernard et al. proposed a voltage-based plasticity model that can explain the location-dependence of the expression of long-term potentiation and long-term depression along the dendrites. They suggest that the combination of presynaptic signaling and the time course of the postsynaptic voltage can predict the outcome of synaptic plasticity at the stimulated synapses. Yates and Scholl investigated how a variety of synaptic inputs produce selective somatic firing. Using a population coding theory, they suggest that heterogeneity in synaptic weight is critical to induce the somatic selectivity based on diverse synaptic input, which matches the functional heterogeneity of dendritic spines in the ferret visual cortex.

A single brain region consists of diverse neuron types that use different subcellular computation algorithms. Moberg and Takahashi focused on how two distinct populations of pyramidal neurons in layer 5 of the somatosensory cortex, intratelencepharic (IT) and extratelencepharic (ET), play their unique roles in sensory processing and behavior. The review highlights the differences in network configurations and physiological and morphological properties between IT and ET neurons, which contribute to their distinct roles in subcellular computation and behavior.

Regional variations in dendritic computations are an important issue in understanding the diversity of subcellular computations, but they are often overlooked. Dendrites of pyramidal neurons in the cortex and the hippocampus express active Na^+^, Ca^2+^, and NMDA receptor conductances, and the clustering of synapses amplifies EPSPs to induce supralinear integration by activating these conductances. Contrarily, this amplification is prevented in the nucleus laminaris (NL) dendrites since their active conductances are weaker than pyramidal cells (Yamada and Kuba). The unique characteristics, including morphology and channel distribution, enable the neurons in NL to encode interaural time differences for sound-source localization.

The current understanding of subcellular computation is mainly about glutamatergic and GABAergic inputs, with a recognition of the crucial role played by the spatial distributions of excitatory and inhibitory synapses in shaping firing specificity (Yamada and Kuba). However, accumulating evidence suggests that neuromodulators also affect subcellular computations. Moberg and Takahashi summarized the effects of individual neuromodulators on excitability and synaptic input in the specific cortical pyramidal neurons (IT and ET). They hypothesized that spatial and temporal dynamics of neuromodulator release play important roles in synaptic integration because neuromodulators change the gain of excitability in cell-type specific manner.

This Research Topic illustrates the diversity of questions, methods, and animal models involved in the study of this field. Computational simulations are also powerful tools to understand subcellular computations as demonstrated in the two original papers (Meissner-Bernard et al. and Yates and Scholl). Our hope is that the information on this Research Topic will be useful for both experimentalists and modelers. We also hope that the papers collected on this Research Topic stimulate researchers in the field and highlight important questions to be addressed in the coming years.

## Author contributions

All authors listed have made a substantial, direct, and intellectual contribution to the work and approved it for publication.

